# Associations Between Disordered Microbial Metabolites and Changes of Neurotransmitters in Depressed Mice

**DOI:** 10.3389/fcimb.2022.906303

**Published:** 2022-05-20

**Authors:** Jing Xie, Ying Wang, Qi Zhong, Shun-jie Bai, Chan-juan Zhou, Tian Tian, Jian-jun Chen

**Affiliations:** ^1^ Department of Endocrinology, The Fourth People’s Hospital of Chongqing, Chongqing University Central Hospital, Chongqing Emergency Medical Center, Chongqing, China; ^2^ Department of Rehabilitation, The Second Affiliated Hospital of Chongqing Medical University, Chongqing, China; ^3^ Institute of Life Sciences, Chongqing Medical University, Chongqing, China; ^4^ Department of Laboratory Medicine, The First Affiliated Hospital of Chongqing Medical University, Chongqing, China; ^5^ Central Laboratory, Yongchuan Hospital of Chongqing Medical University, Chongqing, China; ^6^ Department of Neurology, Guizhou Medical University Affiliated Hospital, Guizhou, China

**Keywords:** depression, amino acids, microbial metabolites, neurotransmitters, prefrontal cortex

## Abstract

**Backgrounds:**

Many pieces of evidence demonstrated that there were close relationships between gut microbiota and depression. However, the specific molecular mechanisms were still unknown. Here, using targeted metabolomics, this study was conducted to explore the relationships between microbial metabolites in feces and neurotransmitters in prefrontal cortex of depressed mice.

**Methods:**

Chronic unpredictable mild stress (CUMS) model of depression was built in this study. Targeted liquid chromatography–mass spectrometry analysis was used to detect the microbial metabolites in feces and neurotransmitters in prefrontal cortex of mice. Both univariate and multivariate statistical analyses were applied to identify the differential microbial metabolites and neurotransmitters and explore relationships between them.

**Results:**

Ninety-eight differential microbial metabolites (mainly belonged to amino acids, fatty acids, and bile acids) and 11 differential neurotransmitters (belonged to tryptophan pathway, GABAergic pathway, and catecholaminergic pathway) were identified. Five affected amino acid–related metabolic pathways were found in depressed mice. The 19 differential microbial metabolites and 10 differential neurotransmitters were found to be significantly correlated with depressive-like behaviors. The two differential neurotransmitters (tyrosine and glutamate) and differential microbial metabolites belonged to amino acids had greater contributions to the overall correlations between microbial metabolites and neurotransmitters. In addition, the significantly decreased L-tyrosine as microbial metabolites and tyrosine as neurotransmitter had the significantly positive correlation (r = 0.681, *p* = 0.0009).

**Conclusions:**

These results indicated that CUMS-induced disturbances of microbial metabolites (especially amino acids) might affect the levels of neurotransmitters in prefrontal cortex and then caused the onset of depression. Our findings could broaden the understanding of how gut microbiota was involved in the onset of depression.

## Introduction

Depression is a common and debilitating neuropsychiatric disorder that affects about 10% of the world population every year (Zhu et al., 2020; Simpson et al., 2021). It causes a high economic burden to individuals and society (Doran and Kinchin, 2019). What is worse is that there is a strong association between depression and suicide (Qiao et al., 2020; Jiang et al., 2021; Balakrishnan et al., 2022). About 800,000 patients with depression in the whole world have suicide behaviors or suicide attempts every year (Wilkowska et al., 2021). Nowadays, many psychotherapeutic and psychotropic treatments have been developed to treat depression, but none of these treatment modalities can completely reverse the multi-factorial pathology of depression (Liu et al., 2021; Methiwala et al., 2021). These phenomena suggest that the currently available theories are not able to systematically and completely elucidate the pathogenesis of depression. Accordingly, it is necessary to identify new insights into the molecular mechanisms of depression, which can be helpful for developing more effective treatment methods.

The balance of gut microbiota is very important for host’s health (Han et al., 2020; Kovács et al., 2021; Liu et al., 2021; Rajput et al., 2021). Studies have reported that the onset and development of many diseases, such as autism and diabetes, have a close relationship with the disturbance of gut microbiota (Khoshkam et al., 2021; Li et al., 2021; Medici Dualib et al., 2021; Xu et al., 2021). In our previous studies, we found that there were significant differences on the gut microbiota compositions in both patients with depression and depressed mice (DM) compared with their respective healthy controls (Zheng et al., 2016; Wu et al., 2020; Bai et al., 2021; Tian et al., 2021). Other researchers also reported the similar results (Naseribafrouei et al., 2014; Jiang et al., 2015). The extant literature indicates that the microbial metabolites may be the key regulators linking the gut microbiota and brain functions (Madison and Kiecolt-Glaser, 2019; Wu et al., 2020; Li et al., 2021). However, the specific mechanisms of action between microbial metabolites and depression are still unclear.

Neurotransmitters, as the chemical messenger, have many important functions in central nervous system (Breitinger et al., 2021; Liu et al., 2021; Yamaguchi et al., 2021). Many pieces of evidence demonstrated that the disturbance of neurotransmitters could be the hallmark of depression (Milak et al., 2016; Zheng et al., 2016; Oh et al., 2020; Afzal et al., 2021). Our group found that a panel consisting of four plasma neurotransmitters could effectively diagnose patients with depression from healthy controls (Pan et al., 2018), and we also observed the disturbance of neurotransmitters in the hippocampus and hypothalamus of DM (Pan et al., 2019; Wang et al., 2020; Wu et al., 2020; Tian et al., 2021). Growing pieces of evidence suggested that there were close relationships between gut microbiota and neurotransmitters (Chen et al., 2021; Huang and Wu, 2021; Wang et al., 2021). Using chronic restraint stress (CRS)–induced depression model, we found the close relationships between disordered microbial metabolites and changed neurotransmitters in the hippocampus and hypothalamus of DM (Wu et al., 2020; Tian et al., 2021). These findings indicated that neurotransmitters might be the bridge between microbial metabolites and depression. Therefore, in this study, using chronic unpredictable mild stress (CUMS) model of depression and targeted metabolomics, we tried to find out how microbial metabolites affected the levels of neurotransmitters in the prefrontal cortex of DM. Our findings will provide some novel clues to explore the pathogenesis of depression.

## Materials and Methods

### Animal Model

Laboratory Animal Center of Chongqing Medical University provided the C57BL/6 male mice (8 weeks of age), which were separately housed under standard conditions. Ethics Committee of Chongqing Medical University reviewed and approved this study (Approval No. 20170301). The experiment was performed according to the National Institutes of Health Guidelines for Animal Research (Guide for the Care and Use of Laboratory Animals, NIH Publication No.8023, revised 1996). After adaption, the mice were randomly assigned into the experiment group and control group. There were 10 mice in each group. The two groups were matched in age: sucrose preference (SPF) and body weight (BW). The experimental group was exposed to CUMS for 4 weeks. The CUMS was conducted according to the procedures in our previous studies (Mao et al., 2017; Gong et al., 2019) (**Supplementary Table 1**). The control group was allowed to obtain food and water freely. The two groups were housed in different rooms to avoid the potential olfactory or acoustic communications.

### Behavioral Tests

The following behavioral tests were conducted in this study: sucrose preference test (SPT), open field test (OFT), and forced swim test (FST). These behavioral tests were conducted according to the procedures in our previous studies (Pan et al., 2018; Pan et al., 2019) (**Supplementary File 1**). The investigators were blinded to the group allocation and outcomes assessment. The two experiment operators were blinded to the group allocation. The following indicators were collected after CUMS to evaluate the depressive-like behaviors (DLBs) in the experiment group: SPF in SPT, center area distance (CD) and center area time (CT) in OFT, and immobility time (IT) in FST. Meanwhile, the BW in the two groups was also collected after CUMS.

### Targeted Microbial Metabolites and Neurotransmitters Detection

Fecal samples and prefrontal cortex were collected from mice in both groups. The sample preparation procedures were conducted according to the procedures in our previous studies (Wu et al., 2020; Zheng et al., 2021). The targeted microbial metabolites and neurotransmitters detection were conducted in Applied Protein Technology Company (Shanghai, China). The detection procedures of microbial metabolites in fecal samples and neurotransmitters in prefrontal cortex using liquid chromatography–mass spectrometry analysis have been reported in our previous studies (Wu et al., 2020; Zheng et al., 2021). The detailed information was described in **Supplementary File 1**.

### Statistical Analysis

Data were expressed in mean ± standard deviation. The Student’s t-test or non-parametric Mann-Whitney U-test was used according to the results of normality test. The orthogonal partial least squares (OPLS) method was used to find out the differential microbial metabolites between DM and control mice (CM). The microbial metabolites with variable importance in projection (VIP) > 1.0 (equivalent to a p-value of less than 0.05) were viewed as the differential microbial metabolites. Then, to identify the potential affected pathways, we used the online software MetaboAnalyst 5.0 to further analyze these differential microbial metabolites. Meanwhile, Pearson correlation analysis was used to identify the potential DLB-related microbial metabolites and neurotransmitters. The relationships between differential neurotransmitters and microbial metabolites were also analyzed. The SPSS 19.0 and R 4.0 were used to do all the analyses, and *p* < 0.05 was considered to be statistically significant. The method of Benjamini and Hochberg’s false discovery rate (FDR) was used here to conduct multiple testing corrections.

## Results

### Behavioral Characteristics in Two Groups

**Figure 1:**
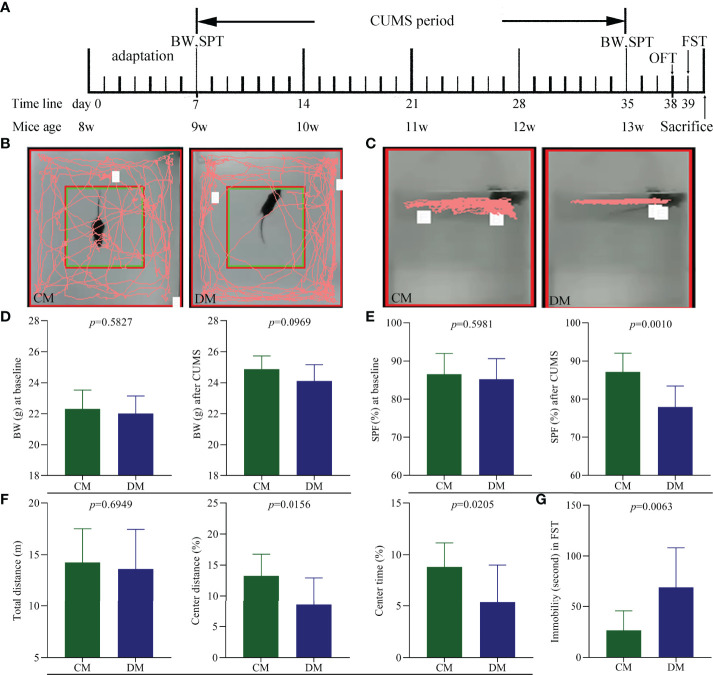
Depressive-like behavior in CUMS-induced depressed mice. **(A)** Diagram of CUMS procedure (n = 10 in each group). Body weight (BW) and sucrose preference test (SPT) were measured after one week adaptation and after weeks of CUMS exposure. Open field test (OFT) and forced swim test (FST) were conducted on day 38 and 39, respectively. All mice were sacrificed on day 40; **(B, C)** the representative trajectories of CM and DM mice in OFT **(B)** and FST **(C)**; **(D)** the two groups had the similar BW at baseline and after CUMS; **(E)** the significant difference on sucrose preference (SPF) (%) was not observed at baseline but found after CUMS between the two groups; **(F)** OFT: the two groups had the similar total distance, but the significant decrease was found in center area distance (%) and center area time in DM; **(G)** FST: the significant increase was found in immobility time (second) in DM. CM, control mice; DM, depressed mice.

Diagram of CUMS procedure was displayed in **Figure 1A**. The representative trajectories of CM and DM in OFT (**Figure 1B**) and FST (**Figure 1C**) were also displayed. There were no significant differences on BW between the two groups at baseline (*p* = 0.5827) and after CUMS (*p* = 0.0969) (**Figure 1D**). The two groups had the similar SPF at baseline (*p* = 0.5981), but after CUMS, the DM had the significant lower SPF compare to CM (*p* = 0.0010, **Figure 1E**). In OFT, the two groups had the similar total distances (*p* = 0.6949), suggesting the comparable motor functions of DM and CM; but both CD (%) (*p* = 0.0156) and CT (%) (*p* = 0.0205) were significantly lower in DM than in CM (**Figure 1F**). Meanwhile, the results of FST showed that the DM had the significantly higher IT compared with CM (*p* = 0.0063, **Figure 1G**). The significant differences on CD, CT, IT, and SPF between the two groups demonstrated that the CUMS successfully induced DLB in mice.

### Differential Microbial Metabolites

**Figure 2:**
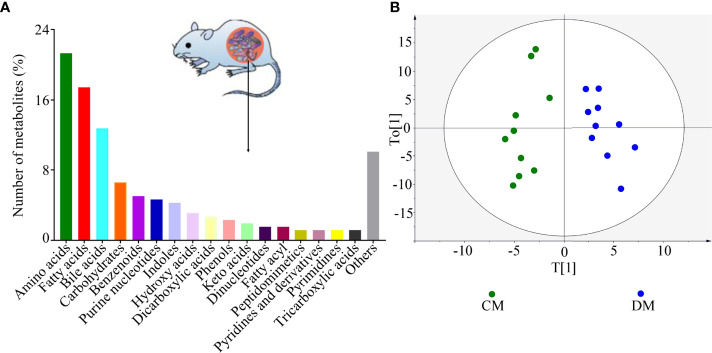
Divergent microbial metabolic phenotypes between DM and CM. **(A)** The identified number of microbial metabolites in each category; **(B)** DM and CM could be obviously separately by the discriminative model built with these identified metabolites, suggesting the divergent microbial metabolic phenotypes between the two groups. CM, control mice; DM, depressed mice.

As shown in **Figure 2A**, the identified microbial metabolites mainly belonged to amino acids (21.32%), fatty acids (17.44%), bile acids (12.79%), carbohydrates (6.59%), benzenoids (5.04%), purine nucleotides (4.65%), and indoles (4.26%). The microbial metabolites were used to build the discriminative model. The results of OPLS model showed that the DM was effectively separated from the CM with no overlap, suggesting the divergent microbial metabolic phenotypes between the two groups (**Figure 2B**). Meanwhile, the results of 399-permutation test (**Supplementary Figure 1**) showed that the built model was valid and not over-fitting. These results demonstrated that the microbial metabolic differences between the two groups were robust.

**Figure 3:**
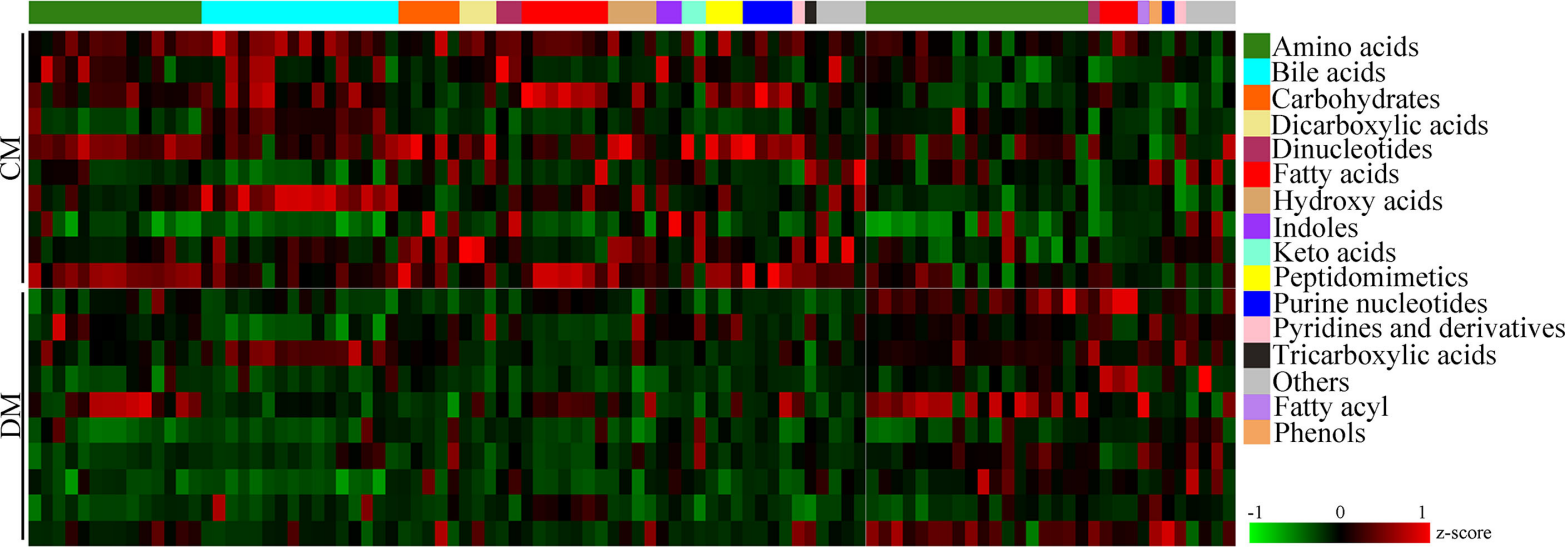
Differential microbial metabolites in DM. The heatmap of the identified differential metabolites showed a consistent clustering pattern within the individual groups. CM, control mice; DM, depressed mice.

According to the corresponding loading plots, the corresponding variables with VIP >1.0 were viewed as the differential microbial metabolites responsible for the discrimination between DM and CM. In total, 98 microbial metabolites with VIP >1.0 were identified in this study; these metabolites mainly belonged to amino acids (n = 32), bile acids (n = 16), and fatty acids (n=10). Compared with CM, the DM was characterized by the lower levels of 68 microbial metabolites, along with the higher levels of 30 microbial metabolites. The detailed information of these differential microbial metabolites was described in **Supplementary Table 2**. The heatmap building with these differential microbial metabolites showed a consistent clustering pattern within the individual groups (**Figure 3**).

### Potential Affected Pathways

**Figure 4:**
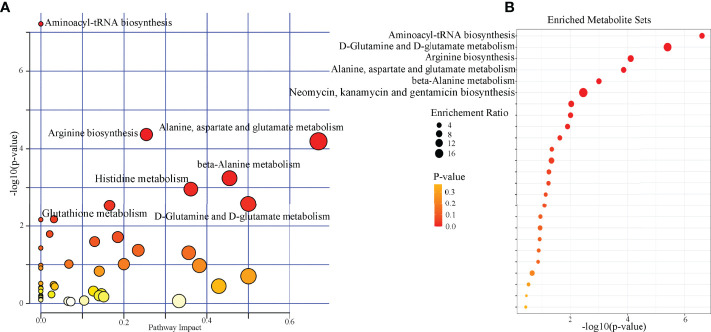
Biological functions analysis of the differential microbial metabolites. **(A)** Pathway analysis showed that seven pathways were significantly affected (p < 0.05, FDR < 0.05); **(B)** six significantly enriched metabolites sets were found (p < 0.05, FDR < 0.05).

Pathway analysis was conducted using the identified differential microbial metabolites. The results showed that seven pathways were significantly affected (aminoacyl-tRNA biosynthesis; arginine biosynthesis; alanine, aspartate, and glutamate metabolism; beta-alanine metabolism; histidine metabolism; D-glutamine and D-glutamate metabolism; and glutathione metabolism) (*p* < 0.05, FDR < 0.05) (**Figure 4A**). Meanwhile, enrichment analysis was conducted using the identified differential microbial metabolites. The results showed that six enriched metabolites sets were identified (aminoacyl-tRNA biosynthesis; D-glutamine and D-glutamate metabolism; arginine biosynthesis; alanine, aspartate, and glutamate metabolism; beta-alanine metabolism; and neomycin, kanamycin, and gentamicin biosynthesis) (*p* < 0.05, FDR < 0.05) (**Figure 4B**). Combining the results of pathway analysis and enrichment analysis, the five affected amino acid–related metabolic pathways in DM were identified: aminoacyl-tRNA biosynthesis; D-glutamine and D-glutamate metabolism; arginine biosynthesis; alanine, aspartate, and glutamate metabolism; and beta-alanine metabolism. The detailed information of these pathways was described in **Supplementary Table 3**.

### DLB-Related Microbial Metabolites

**Figure 5:**
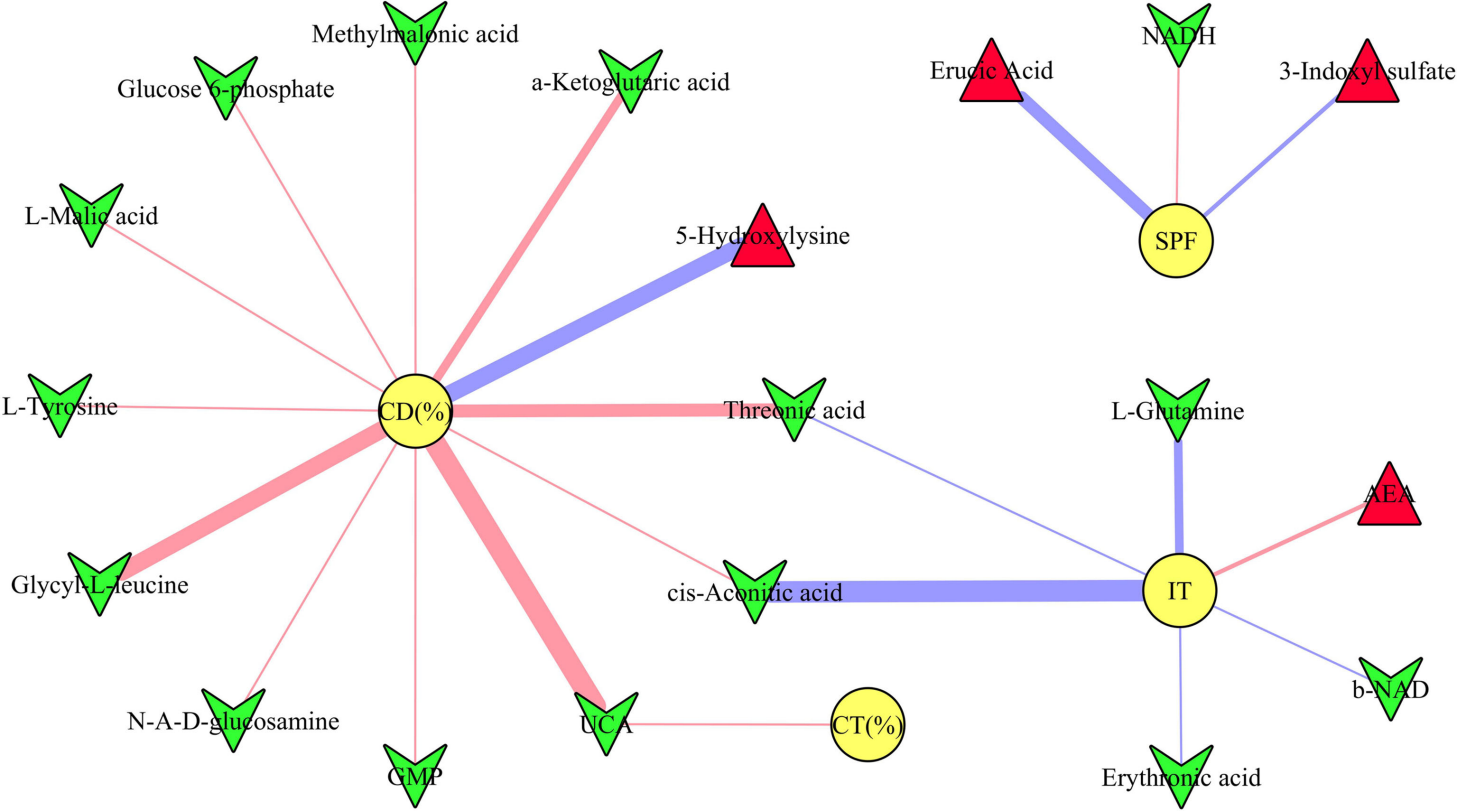
Correlations between depressive-like behavior and differential microbial metabolites. The green inverted triangle represents decreased metabolites, and red triangle represents increased metabolites; red and blue line indicates positive and negative correlation, respectively. The thicker the line represents the greater the correlation coefficient. CD (%), center area distance (%); CT (%), center area time (%); IT, immobility time; SPF, sucrose preference.

The results of Pearson correlation analysis showed that there were 19 differential microbial metabolites significantly correlated with at least one type of DLB (*p*-value ranged from 0.0460 to 0.0019, correlation coefficient less than −0.450 or more than 0.450) (**Figure 5**). These metabolites were ursocholic acid, guanosine monophosphate, methylmalonic acid, glucose 6-phosphate, a-Ketoglutaric acid, L-malic acid, glycyl-L-leucine, threonic acid, 1,4-dihydronicotinamide adenine dinucleotide, b-nicotinamide adenine dinucleotide, erythronic acid, cis-aconitic acid, L-tyrosine, N-acetyl-D-glucosamine, L-glutamine, erucic acid, arachidonoyl ethanolamide, 5-hydroxylysine and 3-indoxyl sulfate. The detailed information of correlation coefficients between differential microbial metabolites and DLB was described in **Supplementary Table 4**.

### Differential Neurotransmitters and Its Relationships With DLB

**Figure 6:**
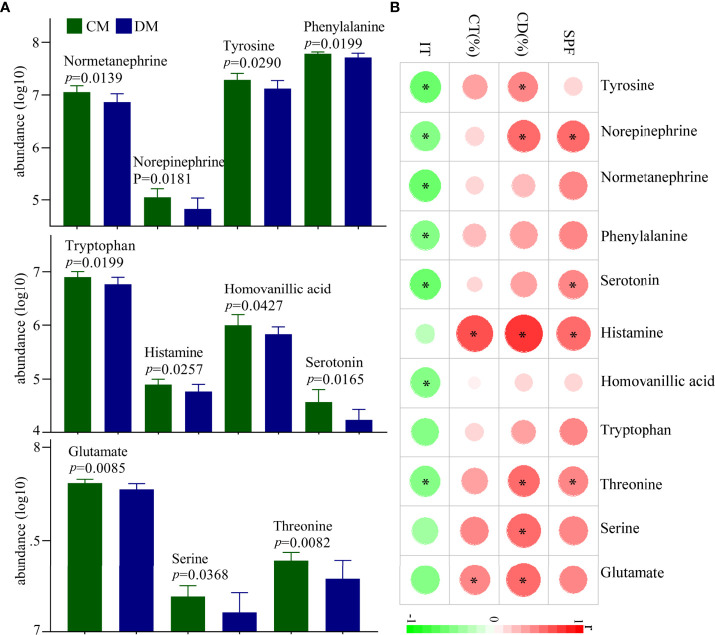
Differential neurotransmitters in the prefrontal cortex of DM. **(A)** Eleven differential neurotransmitters between the two groups; **(B)** the correlations between the differential neurotransmitters and depressive-like behavior. CD (%), center area distance (%); CT(%), center area time (%); IT, immobility time; SPF, sucrose preference; CM, control mice; DM, depressed mice. symbol * indicated p<0.05.

In total, 28 neurotransmitters in prefrontal cortex were identified. Among these neurotransmitters, the levels of eleven neurotransmitters were significantly different between the two groups: glutamate, norepinephrine, normetanephrine, serotonin, histamine, homovanillic acid, tyrosine, phenylalanine, tryptophan, threonine, and serine (**Figure 6A**). The norepinephrine, normetanephrine, tyrosine, and phenylalanine belonged to catecholaminergic pathway; tryptophan, serotonin, histamine, and homovanillic acid belonged to tryptophan pathway; and glutamate, threonine, and serine were closely related to GABAergic pathway. The detailed information of the 28 identified neurotransmitters was described in **Supplementary Table 5**. In addition, we explored the relationships between differential neurotransmitters and DLB (**Figure 6B**). The results showed that the SPF, CD (%), CT (%), and IT were significantly related with four, five, two, and seven neurotransmitters, respectively. The detailed information of correlation coefficients between differential neurotransmitters and DLB was described in **Supplementary Table 6**.

### Correlations Between Differential Microbial Metabolites and Neurotransmitters

**Figure 7:**
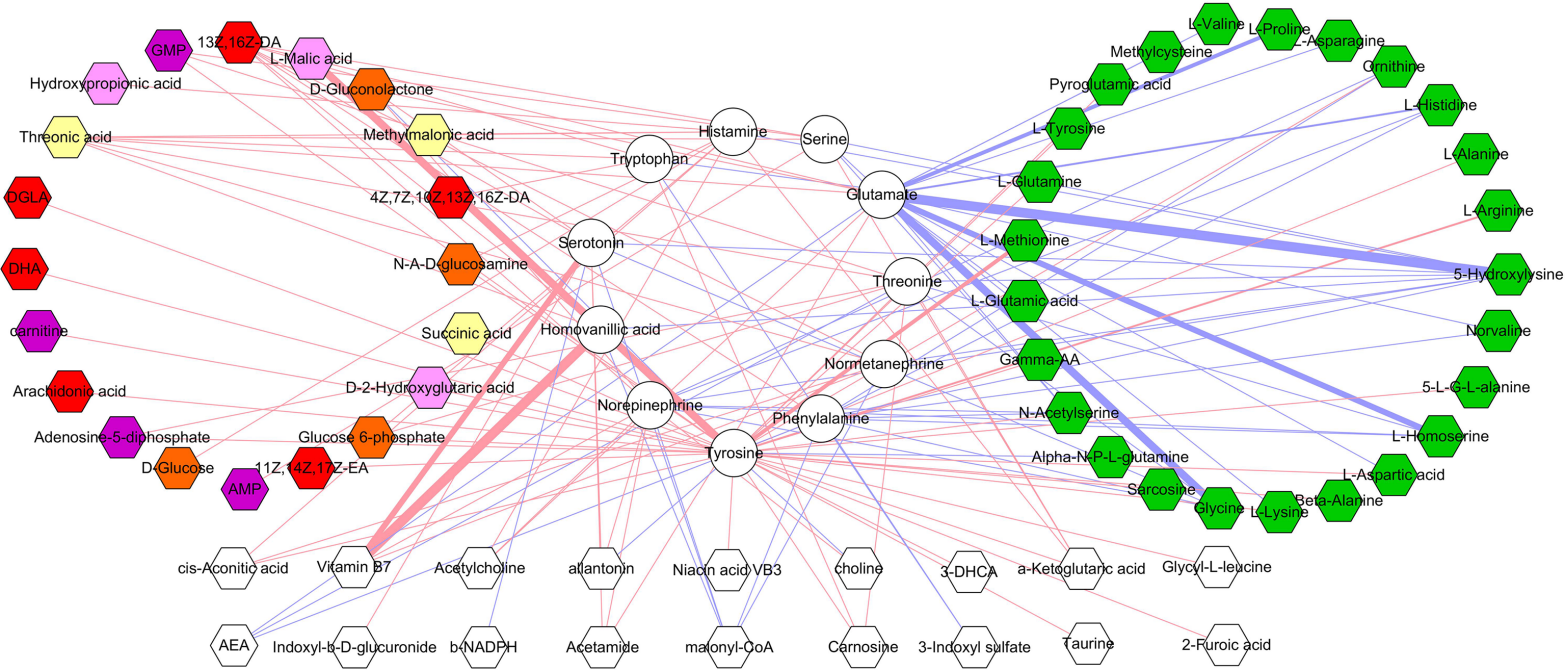
Correlations between differential neurotransmitters and microbial metabolites. The green hexagon represents metabolites belonged to amino acids; red and blue line indicates positive and negative correlation, respectively. The thicker the line, the greater the correlation coefficient.

**Figure 8:**
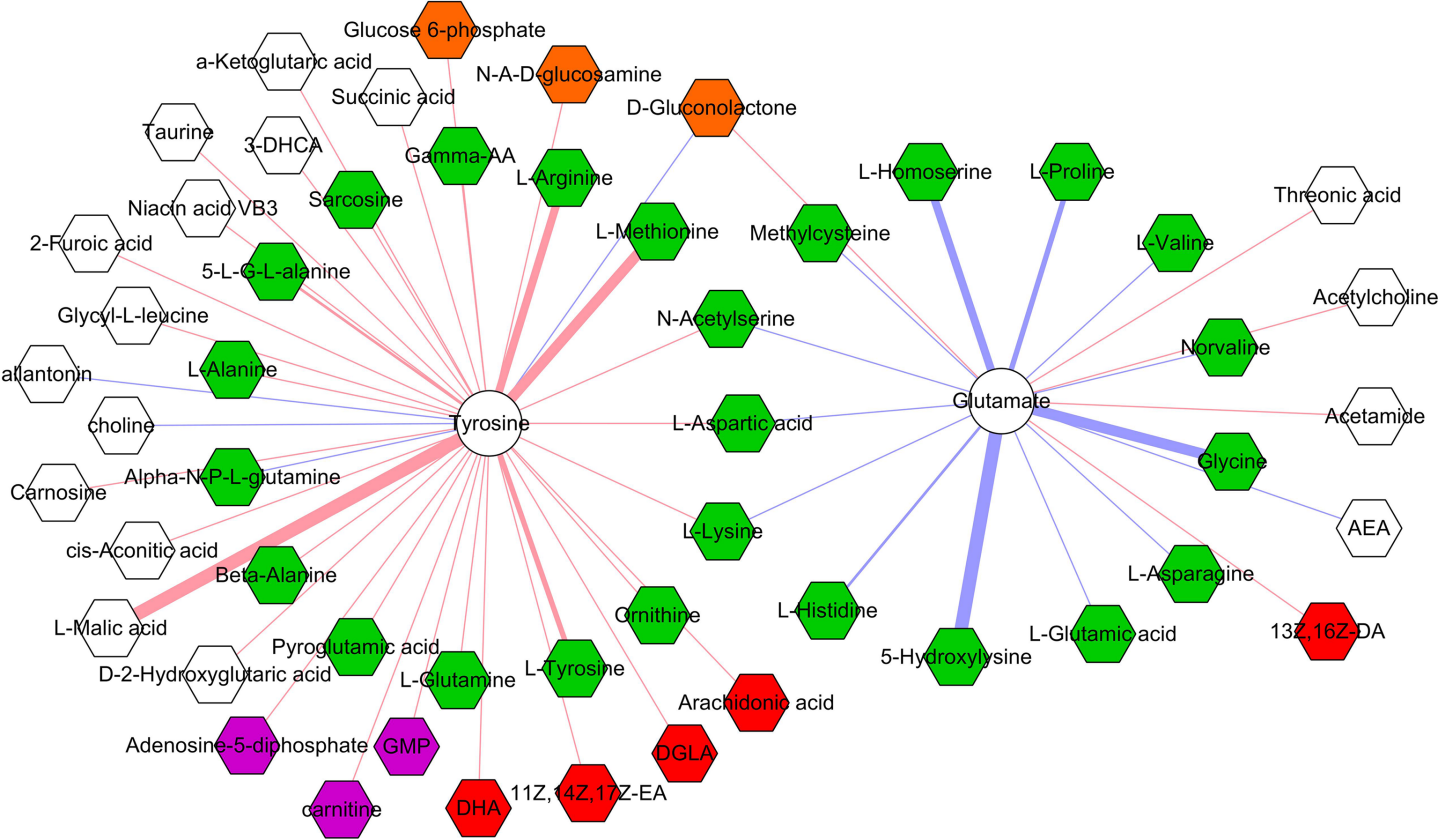
Correlations between two neurotransmitters and 53 microbial metabolites. The green, red, purple, brown, and white hexagons represent metabolites belonged to amino acids, fatty acids, purine nucleotides, carbohydrates, and others, respectively; red and blue line indicates positive and negative correlation, respectively. The thicker the line, the greater the correlation coefficient.

The correlations between differential microbial metabolites and differential neurotransmitters were further studied. We found that the 11 differential neurotransmitters were significantly correlated with 63 differential microbial metabolites (n = 25 belonged to amino acids) (**Figure 7**). Meanwhile, we found that tyrosine and glutamate were significantly correlated with 38 and 19 differential microbial metabolites, respectively (**Figure 8**). These results indicated that the two differential neurotransmitters (tyrosine and glutamate) and differential microbial metabolites belonged to amino acids had greater contributions to the overall correlation. In addition, we observed one interesting clue: the significantly decreased L-tyrosine as microbial metabolites and tyrosine as neurotransmitter had the significantly positive correlation (r = 0.681, *p* = 0.0009).

## Discussion

In this study, using targeted metabolomics, we tried to explore the differences on microbial metabolites in feces and neurotransmitters in prefrontal cortex between DM and CM. The results showed that there were 98 differential microbial metabolites in DM (mainly belonged to amino acids, fatty acids, and bile acids), which were significantly involved in aminoacyl-tRNA biosynthesis; D-glutamine and D-glutamate metabolism; arginine biosynthesis; alanine, aspartate, and glutamate metabolism; and beta-alanine metabolism. The 11 differential neurotransmitters were also identified, which belonged to tryptophan pathway, GABAergic pathway, and catecholaminergic pathway. Meanwhile, we found that there were significant correlations between DLB and differential molecules (microbial metabolites and neurotransmitters). Our findings could provide new insights for further exploring the cross-talk of gut and brain and broaden the understanding of how gut microbiota was involved in the onset of depression.

Amino acids are the important molecules in human body, and its main role is as components of proteins (Das et al., 2020; Farag et al., 2020; Hegazi et al., 2020; Coppolino et al., 2021). However, they are also involved in many important biological functions, such as i) provide energy and ii) serve as neurotransmitters. Our previous studies have identified several significantly changed amino acids in urine and serum of patients with depression compared with healthy controls (Zheng et al., 2013; Zheng et al., 2013; Chen et al., 2018). Other researchers reported that some amino acids might be the potential biomarkers and treatment targets for depression (Baranyi et al., 2016; Koochakpoor et al., 2021). Here, we observed that 32.65% of differential microbial metabolites belonged to amino acids, and identified five affected amino acid–related metabolic pathways. These results indicated that the disordered gut microbiota might play a role in the onset of depression by regulating host’s amino acids.

In our previous study, three neurotransmitters (norepinephrine, serotonin, and 5-hydroxyindoleacetic acid) were found to be significantly decreased in the hypothalamus of CRS-induced DM (Wu et al., 2020). Two of these neurotransmitters (norepinephrine and serotonin) were also significantly decreased here in the prefrontal cortex of DM. In another study, we found that four neurotransmitters (tryptophan, serotonin, indolelactic acid, and 5-hydroxyindoleacetic acid) were significantly decreased in the hippocampus of CRS-induced DM (Tian et al., 2021). Interestingly, the significant decreases of tryptophan and serotonin were also observed here in the prefrontal cortex of DM. To comprehensively understand the changes of neurotransmitters in brain, we significantly expanded the number of neurotransmitters detection in this study compared with our two previous studies (Wu et al., 2020; Tian et al., 2021). Thus, more interesting results were found here, such as the significantly decreased tyrosine in both feces and prefrontal cortex.

**Figure 9:**
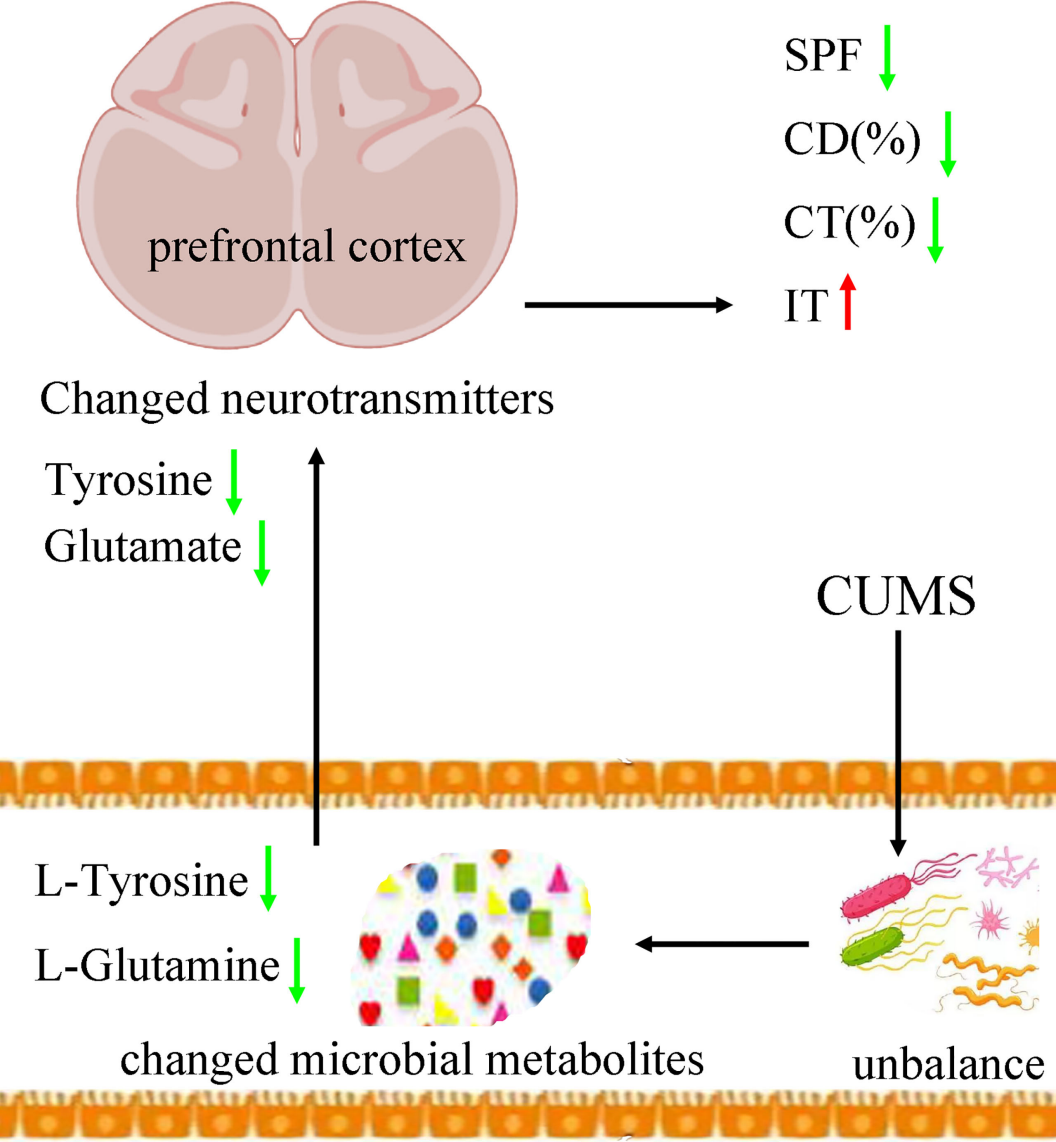
Possible ways in gut–brain axis. CUMS caused the disordered of gut mirobiota, which resulted in the changes of its metabolic products. Then, the differential microbial metabolites, such as L-tyrosine, caused the neurotransmitter disturbance, such as tyrosine and glutamate, which finally induced depressive-like behaviors. CUMS, chronic unpredictable mild stress; CD (%), center area distance (%); CT (%), center area time (%); IT, immobility time; SPF, sucrose preference.

Tyrosine is an amino acid precursor of norepinephrine and dopamine. Both norepinephrine and dopamine have important role in the pathogenesis of depression. A clinical trial showed that acute tyrosine depletion could attenuate the function of dopamine (McLean et al., 2004). Meanwhile, Alabsi et al. reported that tyrosine might be involved in the stress response (Alabsi et al., 2016). On the basis of these results, we deduced that the decreased tyrosine induced by CUMS in feces eventually resulted in the significantly decreased tyrosine in prefrontal cortex, which might be a possible way in gut–brain axis in the onset of depression (**Figure 9**). In addition, our results could also be considered as additional evidence that tyrosine held the promise as the treatment target for depression, which was worthy of further exploring.

Glutamine, the most abundant amino acid in blood, can be directly derived from dietary protein and provide energy for many tissues. It could act as a precursor to glutamate. Although few studies have explored the benefits of glutamine for depression, some researchers still thought that it might be helpful in alleviating depressive symptoms (Son et al., 2018). Son et al. reported that it could produce anti-depressive effects by increasing the levels of glutamate and glutamatergic activity in prefrontal cortex (Son et al., 2018). Our previous study found that the level of glutamine in plasma was significantly decreased in patients with depression compared with healthy controls (Pan et al., 2018). Duman et al. reported that both glutamate and GABA neurotransmitter systems were decreased in some brain regions, such as prefrontal cortex (Duman et al., 2019). Here, we found the significantly decreased level of L-Glutamine in feces and glutamate in prefrontal cortex in DM. On the basis of these results, we suggested that the decreased L-glutamine induced by CUMS in feces eventually resulted in the significantly decreased glutamate in prefrontal cortex, which might be another possible way in gut–brain axis in the onset of depression (**Figure 9**).

Gut microbiota consisting of trillions of bacteria are involved in many biological functions (Huang et al., 2020; Khan et al., 2020; Dordević et al., 2021; Gai et al., 2021; Sun et al., 2021). Tryptophan is an essential amino acid and the precursor for serotonin, and gut microbiota can affect host’s brain function *via* tryptophan metabolism (Gao et al., 2020). Using CRS-induced depression model, we found that “Firmicutes–SCFAs–glycerophospholipids metabolism–tryptophan pathway” might a possible way in the cross-talk of gut and brain (Tian et al., 2021). Meanwhile, we found that some neurotransmitters in tryptophan pathway were significantly changed in patients with depression (Pan et al., 2018). Soh and Walter reported that tryptophan supplementation might be beneficial for certain patients with depression (Soh and Walter, 2011). Here, we observed four significantly decreased neurotransmitters in tryptophan pathway in the prefrontal cortex of DM. However, future studies are still needed to find out whether it is appropriate to treat depression through tryptophan supplementation.

Evidence shows that the disordered GABAergic pathway caused by stress might contribute to the onset of depression (Luscher et al., 2011; Ma et al., 2019). Our group found that the level of glutamate was substantially decreased in the prefrontal cortex of chronic social defeat stress-induced DM (Wang et al., 2016). Many clinical studies reported that patients with depression exhibited significant reductions in GABAergic neurotransmission (Sanacora et al., 2004; Hasler et al., 2007). Meanwhile, previous studies found that catecholaminergic pathway could be activated by stress, and the disordered of this pathway also had close relationships with the onset of depression (Gil et al., 1992; Wang et al., 2016; Hoppmann et al., 2021). In the present study, we found that there were three and four significantly decreased neurotransmitters belonged to GABAergic pathway and catecholaminergic pathway, respectively; and IT was significantly correlated with all the identified neurotransmitters belonged to catecholaminergic pathway. Our results would be helpful for revealing the specific mechanisms of gut microbiota regulating the brain functions.

Several limitations should be mentioned here. First, only feces and prefrontal cortex were collected here; future studies should collect more tissues such as serum and liver, to further identify the potential pathways between gut and brain in depression. Second, although the underlying mechanisms are still unclear, amino acids indeed have a role in maintaining the health of intestinal mucosal barrier. Previous study reported that non-essential amino acids deprivation caused the impairment of barrier function as evidenced by the increase of permeability and decrease of trans-epithelial resistance (Yang et al., 2015). Here, we did not assess whether or not the changes of some amino acids could affect the gut permeability. In our future studies, we will further assess gut permeability to confirm a potential relationship between microbial amino acid and prefrontal cortex neurotransmitter perturbations and explore the effects of tyrosine depletion in changing the gut permeability. Third, in this study, we only analyzed the role of microbial metabolites in our model. Future studies should also consider the role of gut microbiota in regulating the levels of neurotransmitters in brain.

## Conclusion

In conclusion, 98 differential microbial metabolites and 11 differential neurotransmitters in prefrontal cortex of CUMS-induced DM were identified here using targeted metabolomics. These differential metabolites mainly belonged to amino acids, fatty acids, and bile acids; and these differential neurotransmitters belonged to tryptophan pathway, GABAergic pathway, and catecholaminergic pathway. Meanwhile, we found that tyrosine was significantly decreased as microbial metabolites and neurotransmitters, suggesting that tyrosine was worthy of further exploring as the potential treatment target for depression. Our findings provided some novel clues in exploring the pathogenesis of depression.

## Data Availability Statement

The original contributions presented in the study are included in the article/**Supplementary Material**. Further inquiries can be directed to the corresponding author.

## Ethics Statement

The animal study was reviewed and approved by Ethics Committee of Chongqing Medical University.

## Author Contributions

JX and J-jC performed material preparation, data collection, and analysis. JX wrote the first draft of the manuscript. YW, S-jB, C-jZ, TT, and QZ performed model built, software, and methodology. All authors contributed to the article and approved the submitted version.

## Funding

This work was supported by the Natural Science Foundation Project of China (81701360), the Natural Science Foundation of Chongqing (cstc2021jcyj-msxmX0084), the Science and Technology Research Program of Chongqing Municipal Education Commission (Grant No. KJQN202100420), and the Chongqing Yuzhong District Science and Technology Commission (20190115).

## Conflict of Interest

The authors declare that the research was conducted in the absence of any commercial or financial relationships that could be construed as a potential conflict of interest.

## Publisher’s Note

All claims expressed in this article are solely those of the authors and do not necessarily represent those of their affiliated organizations, or those of the publisher, the editors and the reviewers. Any product that may be evaluated in this article, or claim that may be made by its manufacturer, is not guaranteed or endorsed by the publisher.
