# Supplementary material for: Associations Between Disordered Microbial Metabolites and Changes of Neurotransmitters in Depressed Mice
Source: Front Cell Infect Microbiol. 2022 May 20;12:906303. doi: 10.3389/fcimb.2022.906303 (PMC9163491; doi:10.3389/fcimb.2022.906303)
Supplement: Supplementary file 1 [file DataSheet_1.docx]

**Supplementary Methods**

***1. CUMS procedure***

Briefly, the mice in the experiment group were received CUMS for four weeks. Each mouse received one or two different stressors per day, and the same stressor was not allowed to use for two consecutive days. At last, the food and water were deprived for 24 hours before conducting behavioral experiments. The mice in the control group were not disturbed and could freely obtain food and water. The CUMS stressors in every week of CUMS procedure was as following:

**Supplementary Table 1 CUMS stressors in every week of CUMS procedure**

|  | Sunday | Monday | Tuesday | Wednesday | Thursday | Friday | Saturday |
| --- | --- | --- | --- | --- | --- | --- | --- |
| Week1 | CT-24 | SW | LOSL | LOSW | LOTN | WC | FWD |
| Week2 | LOSL | LOTN | 30-IF | SW | FWD | LOSW | CT-24 |
| Week3 | R-4 | FWD | WC | LOTN | SW | LOSL | LOSW |
| Week4 | SW | CT-24 | LOTN | LOSW | R-4 | 30-IF | FWD |

Abbreviations: CT-24, Cages tilted 45°for 24h; SW, swimming in 4℃ water for 5 minutes, wet bedding for 24h; LOSL, lights on overnight and strobe light on for 12h; LOSW, lights on overnight and swimming in 45℃ water for 5 minutes; LOTN, lights on overnight and tail nipped for 1 minute or shaking for 10 minutes; WC, wet cages; FWD, food and water deprivation for 24h; 30-IF, 30 inescapable footshocks; R-4, restraint for 4h.

***2. Behaviors testing methods***

Briefly, in open field test (OFT), we put the mice into the center of box (45 x 45 x 45 cm) to freely explore the box for six minutes. The data produced in last five minutes were collected, including total distance, center time and center distance. In forced swim test (FST), we put the mice into a plexigas cylinder (15 cm diameter x 30 cm height) for six minutes. The plexiglas cylinder was 15 cm diameter and 30 cm height and filled with 18 cm height of water. The temperature of water was 24 ±1°C. The immobility time in last five minutes was collected. In sucrose preference test (SPT), firstly, the mice were trained to adapt to 1% sucrose solution before SPT; secondly, after adaption, the mice were freely access to 1% sucrose solution or water. The consumptions of 1% sucrose solution and water were collected. The sucrose preference was defined as the proportion of 1% sucrose solution consumption in the total liquid consumptions.

***3. Samples preparation***

The procedure of fecal samples for targeted microbial-metabolites was as following: i) after the fecal samples are slowly thawed at 4°C, we added add pre-cooled methanol/acetonitrile/aqueous solution (2:2:1, V/V) into the appropriate amount of sample, and then conducted vortex mixing; ii) low-temperature ultrasound for 30 minutes and standing for 10 minutes (- 20°C), the centrifugation (14000 g, 4°C, 20 minutes) was then conducted; iii) the supernatant was taken and conducted vacuum dry; iv) 100 μL acetonitrile aqueous solution (acetonitrile: water = 1:1, V/V) was added into the dried remains to conduct redissolution and then conducted vortex; and v) after centrifugation (14000 g, 4°C, 15 minutes), the supernatant was injected for later liquid chromatography-mass spectrometry (LC-MS) analysis.

The procedure of prefrontal cortex for targeted neurotransmitters detection was as following: i) added 200 μl ultrapure water (1% formic acid, FA) into the prefrontal cortex, and the mixture were homogenized for three times in a disposable glass tube; ii) 800 μl (1% FA) pre-cooling pure acetonitrile was added into the mixture for vortex mixing; iii) we put the homogenate in the ice bath (−20 °C) for 1 hour incubation, and the obtained protein deposition were induced in an ice bath for 20 min by ultrasound; iv) after centrifugation (14,000 × g, 20 minutes, 4 °C), and the obtained supernatant was transferred into a glass vial to conduct vacuum-drying; v) 100 μl (1% FA) ACN/water (1:1, v/v) were added into the samples to dissolve when conducting mass spectrometry detection; vi) after centrifugation (14,000 × g, 20 minutes, 4 °C), the obtained supernatant was used to identify neurotransmitters using LC-MS system.

***4. LC-MS procedure***

Briefly, the LC-MS procedure of targeted microbial-metabolites was as following. The chromatographic condition: the samples were separated by Agilent 1290 infinity LC ultra high performance liquid chromatography system (UHPLC) HILIC and C18 chromatographic columns; the column temperature of HILIC chromatographic column was 35°C, the flow rate was 0.3ml/min and the injection volume was 2 μl; Mobile phase composition A: 90% water + 2 mM ammonium formate + 10% acetonitrile, B: methanol + 0.4% formic acid; The gradient elution procedure was as follows: 0-1.0 min, 85% B; 1.0-3.0min, B changeed linearly from 85% to 80%; 3.0-4.0 min, 80% B; 4.0-6.0 min, B changed linearly from 80% to 70%; 6.0-10.0 min, B changed linearly from 70% to 50%; 10-12.5 min, B maintained at 50%; 12.5-12.6 min, B changed linearly from 50% to 85%; 12.6-18min, B maintained at 85%. C18 chromatographic column temperature was 40°C, flow rate was 0.4ml/min and injection volume was 2 μl; Mobile phase composition A: water + 5 mM ammonium acetate + 0.2% ammonia, B: 99.5% acetonitrile + 0.5% ammonia; The gradient elution procedure was as follows: 0-5 min, B changed linearly from 5% to 60%; 5-11 min, B changed linearly from 60% to 100%; 11-13 min, B maintained at 100%; 13-13.1 min, B changed linearly from 100% to 5%; 13.1-16min, B maintained at 5%; During the whole analysis process, the samples were placed in a 4°C automatic sampler. In order to avoid the influence caused by the fluctuation of instrument detection signal, the random sequence was used for continuous analysis of samples. QC samples were inserted into the sample queue to monitor and evaluate the stability of the system and the reliability of experimental data. The mass spectrometry conditions: AB 6500 qtrap mass spectrometer was used (AB SCIEX) for mass spectrometry analysis. The ESI source conditions are as follows: weather gas temperature, 350°C; dry gas temperature, 350°C; weather gas flow, 11L/ min; dry gas flow, 10 L/min; capacitive voltage, 4000 V or - 3500 V in positive or negative modes, carefully; nozzle voltage, 500 V; and nebulizer pressure, 30 psi, monitored by multiple reaction monitoring (MRM) mode. We used multiquant or analyst software to extract the peak of MRM raw data to obtain the ratio of peak area of each substance to internal standard peak area. Then, we calculated the content of metabolites according to the standard curve.

Briefly, the LC-MS procedure of targeted neurotransmitters detection was as following. The chromatographic condition: the samples were separated by Agilent 1290 infinity LC ultra high performance liquid chromatography system. Mobile phase composition A: 25 mM ammonium formate aqueous solution containing 0.1% FA, and B was 0.1% FA acetonitrile. The sample was placed in a 4°C automatic sampler, the column temperature was 45°C, and the flow rate was 300 μl/min, injection volume was 2μl. The relevant liquid phase gradient was as follows: 0-18 min, liquid B changed linearly from 90% to 40%; 18-18.1 min, liquid B changed linearly from 40% to 90%; 18.1-23 min, liquid B maintained at 90%. A QC sample was set for a certain number of experimental samples at each interval in the sample queue to detect and evaluate the stability and repeatability of the system; the sample cohort was set up with a standard mixture of neurotransmitters for the correction of chromatographic retention time. Mass spectrometry was performed in positive ion mode with 5500 qtrap mass spectrometer (AB SCIEX). 5500 qtrap ESI source conditions were as follows: source temperature 450°C, ion source gas1 (gas1): 60, ion source gas2 (gas2): 60, curtain gas (cur): 30, ion sapary voltage floating (isvf) 5000 V; MRM mode was used to detect the ion pair to be tested. The chromatographic peak area and retention time were extracted by multiquant software. The standard of neurotransmitters was used to correct the retention time to identify the neurotransmitters.

**Supplementary Results**

***1. Permutation test of OPLS model***

To evaluate whether or not our built OPLS model was over-fitting, such as, the built model was efficient for the training set, but inefficient for predicting the new observations, the 399-permutation test was conducted in this study. As shown in Supplementary Figure 1, the blue regression line of the Q2-points intersected the vertical axis (on the left) below zero (-0.309), suggesting the valid of our built OPLS model.


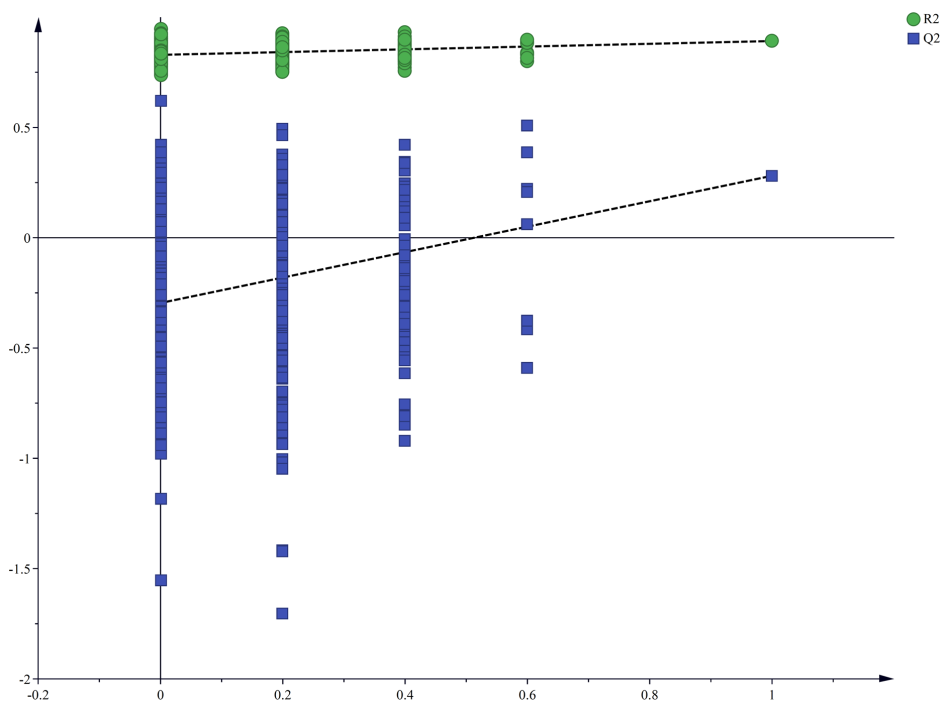


**Supplementary Figure 1 Results of 399-permutation test**

***2. Differential microbial metabolites***

**Supplementary Table 2 Differential microbial metabolites between the two groups**

| **Class** | **Name** | **FC(CM/DM)** | **VIP** |
| --- | --- | --- | --- |
| Amino acids | Gamma-Aminobutyric acid | 1.96 | 1.99 |
| Amino acids | Glycine | 0.77 | 1.78 |
| Amino acids | L-Glutamic acid | 0.97 | 1.06 |
| Amino acids | L-Glutamine | 1.22 | 1.45 |
| Amino acids | Ornithine | 1.15 | 1.03 |
| Amino acids | 2-Phenylglycine | 0.66 | 1.02 |
| Amino acids | 5-Hydroxylysine | 0.49 | 1.90 |
| Amino acids | Alpha-N-Phenylacetyl-L-glutamine | 0.74 | 1.17 |
| Amino acids | Aminoadipic acid | 1.16 | 1.03 |
| Amino acids | Beta-Alanine | 1.17 | 1.27 |
| Amino acids | L-Alanine | 1.18 | 1.28 |
| Amino acids | L-Arginine | 1.13 | 1.29 |
| Amino acids | L-Asparagine | 0.70 | 2.06 |
| Amino acids | L-Aspartic acid | 0.93 | 1.07 |
| Amino acids | L-Histidine | 0.82 | 1.56 |
| Amino acids | L-Lysine | 0.98 | 1.18 |
| Amino acids | L-Methionine | 1.13 | 1.28 |
| Amino acids | L-Proline | 0.89 | 1.31 |
| Amino acids | L-threonine | 0.71 | 1.33 |
| Amino acids | L-Tyrosine | 1.31 | 1.51 |
| Amino acids | L-Valine | 0.87 | 1.33 |
| Amino acids | Methylcysteine | 0.74 | 1.06 |
| Amino acids | N-Acetyl-L-alanine | 0.85 | 1.13 |
| Amino acids | N-Acetylserine | 0.88 | 1.02 |
| Amino acids | Pyroglutamic acid | 1.14 | 1.22 |
| Amino acids | Sarcosine | 1.17 | 1.29 |
| Amino acids | L-Homoserine | 0.69 | 1.79 |
| Amino acids | Norvaline | 0.86 | 1.37 |
| Amino acids | 5-L-Glutamyl-L-alanine | 1.15 | 1.16 |
| Amino acids | L-Thyronine | 0.81 | 1.15 |
| Amino acids | 3-hydroxyl-L-kynurenine | 1.62 | 1.17 |
| Amino acids | Leucyl-Glycine | 1.42 | 1.00 |
| Arylsulfates | 3-Indoxyl sulfate | 0.40 | 1.02 |
| Azoles | Allantoin | 0.89 | 1.03 |
| Bile acids | 3-Dehydrocholic acid (3-DHCA) | 2.11 | 1.56 |
| Bile acids | 7-Dehydrocholic acid (7-DHCA) | 3.79 | 1.86 |
| Bile acids | Apocholic acid | 1.60 | 1.26 |
| Bile acids | Beta-Muricholic acid (b-MCA) | 2.43 | 1.57 |
| Bile acids | Beta-Ursodeoxycholic acid (b-UDCA) | 2.05 | 1.22 |
| Bile acids | Glycolithocholic acid-3-Sulfate | 1.29 | 1.04 |
| Bile acids | Isodeoxycholic acid (IsoDCA) | 2.20 | 1.05 |
| Bile acids | Ursocholic acid (UCA) | 3.40 | 1.35 |
| Bile acids | Chenodeoxycholic acid (CDCA) | 1.95 | 1.21 |
| Bile acids | Cholic acid (CA) | 1.38 | 1.32 |
| Bile acids | Deoxycholic acid (DCA) | 2.41 | 1.66 |
| Bile acids | Glycochenodeoxycholic acid (GCDCA) | 1.47 | 1.12 |
| Bile acids | Hyodeoxycholic acid (HDCA) | 2.44 | 1.68 |
| Bile acids | Murocholic acid (MoCA) | 1.96 | 1.78 |
| Bile acids | Ursodeoxycholic acid (UDCA) | 2.17 | 1.31 |
| Bile acids | Omega-muricholic acid (w-MCA) | 1.62 | 1.70 |
| Carbohydrates | N-Acetyl-D-glucosamine | 1.22 | 1.08 |
| Carbohydrates | D-Gluconolactone | 1.01 | 1.10 |
| Carbohydrates | D-Glucose | 3.03 | 1.48 |
| Carbohydrates | Erythronic acid | 1.59 | 1.00 |
| Carbohydrates | Glucose 6-phosphate | 3.75 | 1.72 |
| Carboximidic acids | Acetamide | 1.60 | 1.47 |
| Dicarboxylic acids | Succinic acid | 2.20 | 1.20 |
| Dicarboxylic acids | Threonic acid | 1.86 | 1.70 |
| Dicarboxylic acids | Methylmalonic acid | 5.73 | 1.51 |
| Dinucleotides | b-Nicotinamide adenine dinucleotide | 1.59 | 1.29 |
| Dinucleotides | Dihydronicotinamide-adenine dinucleotide phosphate(b-NADPH) | 0.56 | 1.30 |
| Dinucleotides | 1,4-Dihydronicotinamide adenine dinucleotide(NADH) | 1.60 | 1.08 |
| Fatty acids | 13Z,16Z-Docosadienoic acid | 1.25 | 1.02 |
| Fatty acids | 4Z,7Z,10Z,13Z,16Z,19Z-Docosahexaenoic acid (DHA) | 1.58 | 1.44 |
| Fatty acids | 4Z,7Z,10Z,13Z,16Z-Docosapentaenoic acid | 2.04 | 1.10 |
| Fatty acids | 7Z,10Z,13Z,16Z-Docosatetraenoic acid | 1.64 | 1.04 |
| Fatty acids | Erucic Acid | 0.71 | 1.46 |
| Fatty acids | Dihomo-gamma-linolenic acid | 1.77 | 1.46 |
| Fatty acids | Palmitoleic Acid | 0.60 | 1.38 |
| Fatty acids | 11Z,14Z,17Z-Eicosatrienoic acid | 1.74 | 1.47 |
| Fatty acids | Arachidonic acid | 1.42 | 1.52 |
| Fatty acids | Palmitelaidic acid | 0.66 | 1.23 |
| Fatty acyl | arachidonoyl ethanolamide | 0.54 | 1.21 |
| Furoic acid | 2-Furoic acid | 0.97 | 1.16 |
| Hydroxy acids | D-2-Hydroxyglutaric acid | 1.00 | 1.06 |
| Hydroxy acids | Hydroxypropionic acid | 1.54 | 1.42 |
| Hydroxy acids | L-Malic acid | 2.36 | 1.67 |
| Hydroxy acids | Lactate (D-lactate) | 1.45 | 1.61 |
| Indoles | Indoxyl-b-D-glucuronide | 1.44 | 1.00 |
| Indoles | 3-Indolepropionic acid | 1.50 | 1.46 |
| Keto acids | a-Ketoglutaric acid | 3.50 | 1.56 |
| Keto acids | Alpha-ketoisovaleric acid | 1.09 | 1.02 |
| Organosulfonic acids and derivatives | Taurine | 2.39 | 1.90 |
| Peptidomimetics | Glycyl-L-leucine | 2.16 | 2.08 |
| Peptidomimetics | Anserine | 1.28 | 1.11 |
| Peptidomimetics | Carnosine | 2.86 | 1.80 |
| Phenols | Epinephrine | 0.60 | 1.11 |
| Purine nucleotides | Adenosine monophosphate | 4.36 | 1.37 |
| Purine nucleotides | Guanosine monophosphate | 32.09 | 1.65 |
| Purine nucleotides | Adenosine-5-diphosphate | 3.68 | 1.40 |
| Purine nucleotides | carnitine | 1.36 | 1.35 |
| Purine nucleotides | Vitamin B9 | 0.67 | 1.00 |
| Pyridines | Vitamin B7 | 1.41 | 1.06 |
| Pyridines and derivatives | malonyl-CoA | 0.68 | 1.12 |
| Pyridines and derivatives | Niacin acid VB3 | 1.40 | 1.29 |
| Quaternary ammonium salts | Acetylcholine | 2.75 | 1.38 |
| Quaternary ammonium salts | choline | 0.87 | 1.02 |
| Tricarboxylic acids | cis-Aconitic acid | 1.45 | 1.15 |

Abbreviations: FC, fold change; CM, control mice; DM, depressed mice. VIP, variable importance in projection

***3. Potential affected pathways***

**Supplementary Table 3 Potential affected pathways**

| **Pathway analysis** |  |  |  |
| --- | --- | --- | --- |
| Pathway name | Number of differential metabolites matched | p-value | FDR |
| Aminoacyl-tRNA biosynthesis | 14^a^ | 6.09E-08 | 5.12E-06 |
| Arginine biosynthesis | 6^b^ | 4.28E-05 | 1.79E-03 |
| Alanine, aspartate and glutamate metabolism | 8^c^ | 6.40E-05 | 1.79E-03 |
| beta-Alanine metabolism | 6^d^ | 5.70E-04 | 1.20E-02 |
| Histidine metabolism | 4^e^ | 1.09E-03 | 1.83E-02 |
| D-Glutamine and D-glutamate metabolism | 3^f^ | 2.67E-03 | 3.50E-02 |
| Glutathione metabolism | 4^g^ | 2.92E-03 | 3.50E-02 |
| **Enrichment analysis** |  |  |  |
| Enriched metabolite set | Number of differential metabolites matched | p-value | FDR |
| Aminoacyl-tRNA biosynthesis | 14^a^ | 2.51E-07 | 2.10E-05 |
| D-Glutamine and D-glutamate metabolism | 3^f^ | 3.93E-06 | 1.65E-04 |
| Arginine biosynthesis | 6^b^ | 7.90E-05 | 2.21E-03 |
| Alanine, aspartate and glutamate metabolism | 8^c^ | 1.39E-04 | 2.92E-03 |
| beta-Alanine metabolism | 6^d^ | 1.01E-03 | 1.70E-02 |
| Neomycin, kanamycin and gentamicin biosynthesis | 2^h^ | 3.54E-03 | 4.95E-02 |

^a^L-Asparagine; L-Histidine; L-Arginine; L-Glutamine; Glycine; L-Aspartic acid; L-Methionine; L-Valine; L-Alanine; L-Lysine; L-Threonine; L-Tyrosine; L-Proline; L-Glutamic acid.

^b^L-Glutamic acid; L-Arginine; L-Aspartic acid; Ornithine; L-Glutamine; Oxoglutaric acid.

^c^L-Aspartic acid; L-Asparagine; L-Alanine; L-Glutamic acid; Gamma-Aminobutyric acid; L-Glutamine; Succinic acid; Oxoglutaric acid.

^d^Hydroxypropionic acid; Malonic semialdehyde; Beta-Alanine; L-Aspartic acid; Carnosine; L-Histidine.

L-Glutamate; L-Histidine; Carnosine; L-Aspartate

^f^L-Glutamic acid; L-Glutamine; Oxoglutaric acid.

^g^Glycine; L-Glutamic acid; Pyroglutamic acid; Ornithine.

^h^D-Glucose; Glucose 6-phosphate

***4. Correlations between differential microbial metabolites and DLB***

**Supplementary Table 4** **Correlations between differential microbial metabolites and DLB**

| **DLB** | **Metabolites** | **r** | **p-value** |
| --- | --- | --- | --- |
| SPF | Erucic Acid | -0.474 | 0.0349 |
| SPF | 3-Indoxyl sulfate | -0.471 | 0.0363 |
| SPF | NADH | 0.463 | 0.0398 |
| CD(%) | 5-Hydroxylysine | -0.65 | 0.0019 |
| CD(%) | UCA | 0.633 | 0.0027 |
| CD(%) | Glycyl-L-leucine | 0.62 | 0.0036 |
| CD(%) | Threonic acid | 0.608 | 0.0044 |
| CD(%) | a-Ketoglutaric acid | 0.568 | 0.009 |
| CD(%) | N-A-D-glucosamine | 0.487 | 0.0294 |
| CD(%) | cis-Aconitic acid | 0.469 | 0.0368 |
| CD(%) | L-Tyrosine | 0.46 | 0.041 |
| CD(%) | GMP | 0.458 | 0.0424 |
| CD(%) | L-Malic acid | 0.454 | 0.0444 |
| CD(%) | Glucose 6-phosphate | 0.453 | 0.0447 |
| CD(%) | Methylmalonic acid | 0.45 | 0.0465 |
| CT(%) | UCA | 0.497 | 0.0257 |
| IT | cis-Aconitic acid | -0.651 | 0.0018 |
| IT | AEA | 0.558 | 0.0105 |
| IT | L-Glutamine | -0.472 | 0.0355 |
| IT | Erythronic acid | -0.468 | 0.0374 |
| IT | b-NAD | -0.457 | 0.0428 |
| IT | Threonic acid | -0.451 | 0.0461 |

Abbreviations: DLB, depressive-like behaviors; SPF, sucrose preference; CD, center distance; CT, center time; IT, immobility time.

***5. The 28 identified neurotransmitters in prefrontal cortex***

**Supplementary Table 5** **The 28 identified neurotransmitters in prefrontal cortex**

| **Neurotransmitters** | **FC(CM/DM)** | **p-value** |
| --- | --- | --- |
| Glutamate | 1.08 | 0.0086 |
| Norepinephrine | 1.63 | 0.0182 |
| Normetanephrine | 1.51 | 0.0139 |
| Serotonin | 2.3 | 0.0165 |
| Histamine | 1.32 | 0.0257 |
| Homovanillic acid | 1.53 | 0.0427 |
| Tyrosine | 1.43 | 0.0291 |
| Phenylalanine | 1.17 | 0.0199 |
| Tryptophan | 1.34 | 0.0199 |
| Threonine | 1.24 | 0.0082 |
| Serine | 1.2 | 0.0369 |
| Glutamine | 1.08 | 0.0595 |
| 4-Aminobutyric acid | 1.12 | 0.1354 |
| Dopamine | 1.94 | 0.1028 |
| 3-Methoxytyramine hydrochloride | 1.21 | 0.1979 |
| Tyramine | 1.83 | 0.3901 |
| 5-Hydroxyindole-3-acetic acid | 1.25 | 0.1951 |
| 5-Hydroxy-L-tryptophan | 1.31 | 0.1026 |
| Arginine | 1.24 | 0.0616 |
| rac 4-Hydroxy-3-methoxyphenylethylene Glycol | 1.11 | 0.3321 |
| 3,4-Dihydroxyphenylethyleneglyco | 1.06 | 0.6909 |
| 3,4-Dihydroxymandelic acid | 1.18 | 0.4358 |
| 3-Methoxy-4-hydroxymandelate | 0.91 | 0.6222 |
| 3,4-Dihydroxyphenylacetate | 1.24 | 0.1137 |
| N-acetyl serotonin | 1.32 | 0.094 |
| Ornithine | 1.31 | 0.0615 |
| Ethanolamine | 1.08 | 0.0836 |
| 5-Methoxytryptamine | 1.46 | 0.074 |

Abbreviations: FC, fold change; CM, control mice; DM, depressed mice.

***6. Correlations between differential neurotransmitters and DLB***

**Supplementary Table 6 Correlations between differential neurotransmitters and DLB**

| **DLB** | **Neurotransmitter** | **r** | **p-value** |
| --- | --- | --- | --- |
| SPF | Histamine | 0.583 | 0.007 |
| SPF | Norepinephrine | 0.523 | 0.0179 |
| SPF | Threonine | 0.462 | 0.0404 |
| SPF | Serotonin | 0.459 | 0.042 |
| CD(%) | Histamine | 0.728 | 0.0003 |
| CD(%) | Glutamate | 0.598 | 0.0053 |
| CD(%) | Serine | 0.587 | 0.0065 |
| CD(%) | Norepinephrine | 0.551 | 0.0118 |
| CD(%) | Threonine | 0.503 | 0.0239 |
| CT(%) | Histamine | 0.673 | 0.0011 |
| CT(%) | Glutamate | 0.474 | 0.0346 |
| IT | Normetanephrine | -0.533 | 0.0155 |
| IT | Tyrosine | -0.532 | 0.0158 |
| IT | Serotonin | -0.512 | 0.021 |
| IT | Threonine | -0.483 | 0.031 |
| IT | Norepinephrine | -0.481 | 0.0318 |
| IT | Homovanillic acid | -0.481 | 0.0317 |
| IT | Phenylalanine | -0.453 | 0.0448 |
